# The Role of Obesity in Early and Long-Term Outcomes after Surgical Excision of Lung Oligometastases from Colorectal Cancer

**DOI:** 10.3390/jcm9113566

**Published:** 2020-11-05

**Authors:** Francesco Londero, Orlando Parise, William Grossi, Angelo Morelli, Gianluca Masullo, Michele Bartoletti, Cecilia Tetta, Ugolino Livi, Jos G. Maessen, Sandro Gelsomino

**Affiliations:** 1 Department of Cardiothoracic Surgery, S. Maria della Misericordia University Hospital, 33100 Udine, Italy; francesco.londero@asufc.sanita.fvg.it (F.L.); william.grossi@asufc.sanita.fvg.it (W.G.); angelo.morelli@asufc.sanita.fvg.it (A.M.); gianuca.masullo@asufc.sanita.fvg.it (G.M.); ugo.livi@asufc.sanita.fvg.it (U.L.); 2 Cardiovascular Research Institute, Maastricht University, 6229HX Maastricht, The Netherlands; o.parise@icloud.com (O.P.); j.g.maessen@mumc.nl (J.G.M.); 3 Medical Oncology Department, National Cancer Institute, 33081 Aviano, Italy; michelebarto89@gmail.com; 4 Radiology Department, Rizzoli Institute, 40136 Bologna, Italy; cecilia.tetta@ior.it

**Keywords:** obesity, lung, oligometastases, surgery, colorectal cancer

## Abstract

Obesity correlates with better outcomes in many neoplastic conditions. The aim of this study was to assess its role in the prognosis and morbidity of patients submitted to resection of lung oligometastases from colorectal cancer. Seventy-six patients undergoing a first pulmonary metastasectomy were retrospectively included in the study. Seventeen (22.3%) were obese (body mass index (BMI) >30 kg/m^2^). Assessed outcomes were overall survival, time to recurrence, and incidence of post-operative complications. Median follow-up was 33 months (IQR 16–53). At follow-up, 37 patients (48.6%) died, whereas 39 (51.4%) were alive. A significant difference was found in the 3-year overall survival (obese 80% vs. non-obese 56.8%, *p* = 0.035). Competing risk analysis shows that the cumulative incidence of recurrence was not different between the two groups. Multivariate analysis reveals that the number of metastases (*p* = 0.028), post-operative pneumonia (*p* = 0.042), and DFS (*p* = 0.007) were significant predictors of death. Competing risk regression shows that no independent risk factor for recurrence has been identified. The complication rate was not different between the two groups (17.6% vs. 13.6%, *p* = 0.70). Obesity is a positive prognostic factor for survival after pulmonary metastasectomy for colorectal cancer. Overweight patients do not experience more post-operative complications. Our results need to be confirmed by large multicenter studies.

## 1. Introduction

The prevalence of overweight and obesity in Europe has been constantly rising in the last several decades [1]. Even though higher values of Body Mass Index (BMI) seem to be associated with an increased risk of developing cancer in several organs [2], many articles report improved survival rates in overweight patients with a neoplastic condition [3], in what has been described as “the obesity paradox” [4]. Nonetheless, evidence is controversial and a recent pooled analysis confirmed high BMI to be a positive prognostic factor only in the male sex [5]. The experience in the context of lung oligometastases seems to remark a potential protective effect of obesity over cancer progression, but evidence is still limited [6]. On the other hand, the impact of obesity on the incidence of post-operative complications in this subgroup of patients has never been specifically investigated. The objective of this study was to assess BMI as a potential prognostic factor and a predictive factor of early post-operative outcome in a population of patients undergoing pulmonary metastasectomy for oligometastases from colorectal cancer.

## 2. Materials and Methods

This paper was structured according to the Strengthening the Reporting of Observational Studies in Epidemiology (STROBE) statement [7].

Approval of the study was waived by the Ethical Committee due to the retrospective nature of its design, according to National Laws regulating observational retrospective studies (Italian law nr.11960, released on 13/07/2004). However, patients gave their written informed consent for the treatment of their data for scientific purposes.

### 2.1. Patient Population

We retrospectively reviewed all clinical records from patients who underwent lung metastasectomy with curative intent for metastases from colorectal cancer (CRC) between January 2005 and December 2017 in a single center (S. Maria della Misericordia University Hospital, Udine, Italy). Inclusion criteria for the study were as follows:

(1) Previous complete excision of the primary tumor and without other localizations of disease, or extrathoracic deposits amenable to local aggressive treatments;
(2) No history of previous excision of lung metastases;
(3) No macroscopic residual disease following metastasectomy;
(4) Confirmation of the metastatic nature of excised nodules through histology report.

Patients were divided into two groups: patients with a BMI >30 kg/m^2^ or greater (obese) and patients with a BMI <30 kg/m^2^ (non-obese) according to the definitions of the World Health Organization (WHO) [8]. Biometric parameters were measured at admission, the day before the planned metastasectomy. During the time interval, 76 patients underwent pulmonary resection with curative intent for oligometastases from CRC. Patients’ characteristics are reported in Table 1.

The primary outcomes we investigated were overall survival (OS) and disease-free survival (DFS). OS was defined as the time interval between metastasectomy and death or censored event, whereas DFS was defined as the time interval between metastasectomy and tumor recurrence or censored event. The secondary outcome assessed was incidence of post-operative complications.

### 2.2. Surgery

Indication for surgery was discussed at our local multidisciplinary team meeting involving medical and radiation oncologists, surgeons, radiologists, chest physicians, and pathologists, and offered on the basis of oncological and functional aspects. In addition to the general criteria that suggest an indication for ablative treatments—controlled primary tumor and extrapulmonary localizations amenable to local aggressive treatments—selection factors for surgery were as follows: (1) pulmonary oligometastases had to be considered suitable for complete resection; (2) predicted post-operative forced expiratory volume in the first second (ppo-FEV1) and diffusion lung capacity for carbon monoxide (ppo-DLCO) had to be >40%; (3) patients were expected to recover with a good quality of life.

Details on surgical procedure are reported in Table 2. Both open and minimally invasive approaches were employed. Sub-lobar resections were usually performed for small peripheral nodules, whereas anatomical major resections were required for large or central lesions or multiple nodules confined to one lobe. One patient underwent a laser tumorectomy and was included in the wedge resection group as a non-anatomical resection. Bilateral deposits were approached with bilateral synchronous or staged procedures, being the first procedure used as the starting point for survival analysis.

We collected data on pre-operative patients’ features (age, gender, American Society of Anesthesiology (ASA) class, and comorbidities), oncologic aspects (disease-free interval (DFI), primary tumor stage, adjuvant/neoadjuvant treatment, and the number of pulmonary metastases), surgical modalities (approach and kind of resection), and outcomes (length of follow-up, pattern of recurrence and survival, and post-operative complications). DFI was defined as the time interval between treatment of the primary tumor and the discovery of metastases.

### 2.3. Statistical Analysis

The normality of distribution was assessed using the Kolmogorov-Smirnov test. Continuous data were summarized as mean and standard deviation or the median and 25th to 75th percentiles in the case of non-normal distributions. Categorical variables were reported as counts and percentages. Comparisons were carried out using the Fisher’s exact test and the McNemar test where appropriate.

The Kaplan-Meier method and log-rank test were used for survival analysis. A Cox regression model was used to estimate predictors of death. The proportional hazard assumption was confirmed by the use of Schoenfeld residuals. Cumulative incidence curves were used to graphically depict tumor recurrence, and statistical significance was tested with the Gray test. A competing risk analysis was used to avoid overestimation of the incidence of recurrence. Cut-offs were determined by receiver operating characteristic (ROC) curve analysis as the optimal threshold for predicting death and tumor recurrence. We validated the results using the bootstrap method (1000 iterations). Furthermore, the effect of main predictors was tested using multivariable analysis. R software, release 3.2.3 (R Foundation for Statistical Computing, Wien, Austria), with the “survival” and “cmprsk” packages, was utilized. Significance for hypothesis testing was set at the 0.05 two-tailed level.

## 3. Results

### 3.1. Main Outcomes

Overall, seven patients (9.2%) had bilateral resections, without any significant difference between the two groups (*p* > 0.99, Table 1). Synchronous metastases were detected in 9.2% of patients with no difference between obese and non-obese patients. Lymphadenectomy, by means of both lobe-specific nodal sampling and systematic dissection, was performed according to first operator choice in 55.3% of cases, with no significant difference in the two groups (BMI >30 kg/m^2^: 76.5%; BMI <30 kg/m^2^: 49.1%; *p* = 0.06). Only one patient in the obese group had evidence of involved lymph nodes at the time of the final histology report (*p* = 0.22).

Early and long-term results are reported in Table 3.

**Figure 1:**
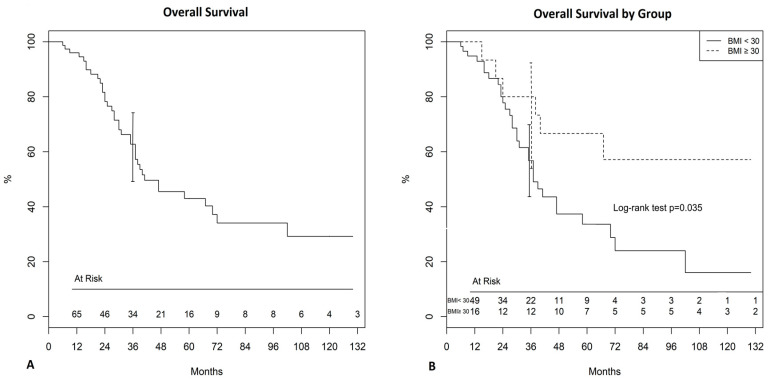
(**A**) Overall survival for the whole population. (**B**) Overall survival by group (obese, BMI >30, and non-obese, BMI <30).

**Figure 2:**
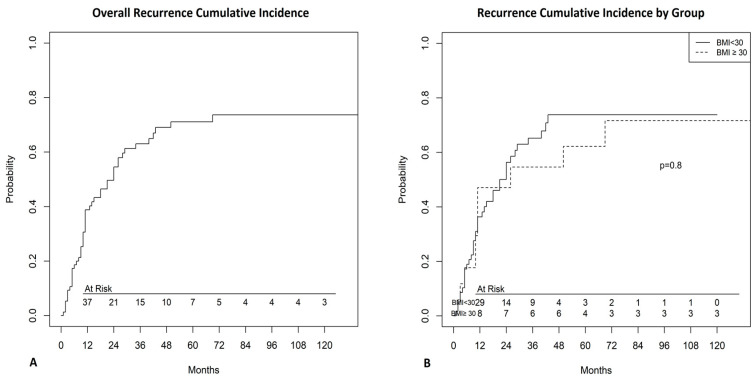
(**A**) Cumulative incidence of recurrence for the whole population. (**B**) Cumulative incidence of recurrence by group (Obese, BMI >30 and non-obese, BMI <30).

At a median follow-up (100% complete) of 33 months (IQR: 16–53 months, range 1–155), 37 patients (48.6%) died, whereas 39 (51.4%) were still alive. A significant difference in overall survival (OS) at 3 years was found between obese and non-obese patients (80% [50–93.1%] vs 56.8%, [41.1–69.7%] *p* = 0.035, Figure 1). Cumulative incidence of recurrence at 3 years was not significantly different between the two groups (obese 43.4% [34.4–80.8%] vs non-obese 68.1% [54.7–80.9%], *p* = 0.8, Figure 2).

In the whole population 14.5% of patients experienced adverse events after surgery and no difference was encountered in the incidence of post-operative complications between obese and non-obese patients.

### 3.2. Predictors of Outcomes

Based on multivariate analysis, obesity (*p* = 0.031), the number of metastases (*p* = 0.028), post-operative pneumonia (*p* = 0.042), and DFS (*p* = 0.007) were found to be significant predictors of death.

Using a ROC curve, the DFS cut-off was 31.5 months (AUC 0.44 [0.35–0.53]) and the number of metastases cut-off was 1.5 (AUC 0.60 [0.47–0.73]). Competing risk regression shows that no independent risk factor for recurrence was identified.

## 4. Discussion

The definition of “obesity paradox” dates back to 2002 and was initially described in the context of cardiovascular disease [9]. Since then, several articles have focused on this issue in the field of oncology, highlighting a more indolent course of disease in overweight patients [3,5,10,11]. This is consistent with the main finding of our study, the significant difference in overall survival at 3 years between the two groups after pulmonary metastasectomy. Obesity was also found, through multivariate analysis, to be an independent risk factor for death. Our results are in accordance with the only other article assessing the role of obesity and overweight on survival after pulmonary metastasectomy [6]: the authors analyzed a population of patients submitted to resection of oligometastases from several primary tumors and found a difference both in OS and DFI, suggesting that obesity induces a slower progression of neoplastic disease after resection of the primary tumor. Quite interestingly, their results were not affected by the lower percentage of radical resections in the group of obese patients [6]. These results are in line with a recent large retrospective study on patients with metastatic cancer treated with radiotherapy, where Tsang and colleagues found overweight and obesity to be independent predictors of better OS [3].

BMI is certainly not the best proxy to express the nutritional status of patients: it does not discriminate the skeletal muscle composition and the adiposity of the body and does not take into account the variations that may occur during the natural history of disease [12]. However, it is easily measurable and showed a good correlation with morbidity and mortality [12]. Patients with advanced cancer usually experience weight loss that is mainly an expression of skeletal muscle consumption, a condition known as “sarcopenia” [4]. Recently, several authors have focused their attention on the role of body composition, measured by impedance analysis [13] or retrospective review of cross-sectional images [4], highlighting the detrimental role of sarcopenia in the clinical course of neoplastic patients.

While many publications contributed to understanding the relationship between obesity and the increased risk of cancer [14], the biological mechanisms behind the paradoxical relationship between body mass and the outcome of many neoplastic conditions are still not fully understood [15]. A possible explanation of this phenomenon has been identified at a molecular level in the microenvironment of cytokines expressed by the body: interleukin-6 (IL-6) is a proinflammatory cytokine that has been shown to induce both weight loss and tumor progression [16,17] and is usually found overexpressed in patients with advanced cancer [16]. Other studies have highlighted the prognostic role of serum leptin and adiponectin in patients with lung cancer [18,19]. The retrospective design of our study did not allow us to perform a precise body mass composition assessment or a measurement of circulating cytokines, but this will be an objective of future research.

Another important finding in our study is that the number of metastases, the development of post-operative pneumonia, and DFS resulted independent predictors of death. The presence of multiple metastases has been demonstrated to be a risk factor for worse prognosis in several previous studies [20,21,22]. The importance of the number of nodules has nonetheless been described in a systematic review of literature on the results of surgical resection of pulmonary metastases from colorectal cancer, where the presence of multiple nodules has been identified as one of the most significant risk factors for death [23]. Therefore, our opinion is that patients presenting with multiple lung metastases from CRC should carefully be considered for resection, notably when other negative prognostic factors coexist.

In our experience, the development of post-operative pneumonia appeared to be an independent negative prognostic factor for survival. The development of post-operative complications has already been demonstrated to correlate with a worse prognosis in a series of patients undergoing resection of pulmonary metastases from head and neck district tumors [24]. This finding has been confirmed in a large series of patients undergoing resection of primary colorectal cancer [25], suggesting a close relationship between inflammation and cancer promotion. The biological basis behind this phenomenon remains uncertain, but it has been theorized that an “innate immune system distraction” mechanism [26] might play a relevant role: the immune system of patients developing an infectious post-operative complication, which normally plays a key role in cancer surveillance and inhibition, may be diverted to the healing of the affected site, therefore allowing uninhibited tumor cell progression [26,27]. This evidence might lead clinicians to pose a surgical indication only in patients with a low risk of post-operative morbidity: in our study, the duration of in-hospital stays and the incidence of post-operative averse events were not significantly different between the two groups, although patients with a BMI >30 kg/m^2^ had a significantly higher prevalence of diabetes and hypertension, which might have predisposed them to an increased risk of post-operative complications such as cardiovascular events and infections. To our knowledge, this study is the first to assess the impact of obesity on the incidence of post-operative complications after pulmonary metastasectomy. In a large series of patients submitted to the surgical resection of liver metastases from CRC, the authors described an increased incidence of adverse events after surgery in overweight patients, mainly due to pulmonary complications [28]. In contrast with this evidence, in a large prospective study on patients undergoing general surgery intervention, obesity was found to determine a mild increased risk of wound infection only, whereas the incidence of major events did not differ from normal weight patients [29]. Increased body weight has an important impact on the respiratory physiology: dynamic lung volumes are reduced [30], and atelectasis persists longer in morbidly obese patients [31], thus leading to a potential increased risk of pulmonary complications [32]. This effect has been confirmed in a surgical series of patients submitted to lung resection for several conditions: obese patients developed a higher increase in alveolar-arterial oxygen difference and hypoxia [33], even though clinically relevant consequences did not occur more frequently among obese patients. The experience derived from subjects undergoing lung resection for primary lung cancer shows conflicting results: Dhakal and colleagues reported no difference in the incidence of post-operative adverse events after anatomical lung resection between obese and normal weight patients [34], and Smith and colleagues demonstrated a slightly higher incidence of respiratory complications in obese patients, albeit without reaching statistical significance [35]. However, a protective effect of obesity on post-operative morbidity and early mortality has been confirmed in a recent meta-analysis on patients receiving lung cancer surgery [36]. BMI seems to play a more relevant role in the case of extensive surgical resection: Petrella and colleagues showed how a BMI >25 kg/m^2^ is a significant risk factor for the development of post-operative complications after pneumonectomy for lung cancer [37].

Therefore, apart from patients proposed for extensive pulmonary resections, obesity may not be seen as a contraindication for surgery or a risk factor for adverse events in the post-operative phase.

## 5. Conclusions

Obese patients undergoing pulmonary metastasectomy with radical intent have a better overall survival compared to normal weight and underweight patients. The incidence of post-operative complications is not more frequent in obese patients. Further research is necessary to confirm our results and identify more accurate prognostic indicators.

## 6. Limitations

This study has some limitations. The retrospective design of the study might carry several biases, and the use of BMI as an indicator of body mass instead of a precise body composition may have underestimated the role of more precise indicators such as skeletal muscle mass. Nonetheless, this study did not take into consideration the variation of weight that patients may have been subjected to during the course of disease. Moreover, although survival analysis was re-weighted by the population number, the low sample size may carry several biases and reduce the reliability of our results.

## Figures and Tables

**Table 1:** Patients and tumor’s characteristics.

Population	All	BMI ≥30	BMI <30	*p*
	76 (100)	17 (22.3)	59 (77.7)	
Gender: Male	49 (64.5)	12 (70.6)	37 (62.7)	0.77
Age at Surgery	67 (IQR 62–70)	65 (IQR 59–68)	69 (IQR 62–73)	0.1
ASA				
2	48 (63.2)	9 (52.9)	39 (66.1)	0.39
3	28 (36.8)	8 (47.1)	20 (33.9)	
Comorbidities				
Coronaropathy	6 (7.9)	1 (5.9)	5 (8.5)	>0.99
Arrhythmia	7 (9.2)	2 (11.7)	5 (8.5)	0.64
Hypertension	26 (34.2)	11 (64.7)	15 (25.4)	0.004
Other Cancers	11 (14.5)	2 (11.8)	9 (15.2)	>0.99
Lung Disease	5 (6.6)	1 (5.9)	4 (6.8)	>0.99
Diabetes	5 (6.6)	4 (23.5)	1 (1.7)	0.008
Chronic Renal Failure	1 (1.3)	0	1 (1.7)	>0.99
Liver Disease	5 (6.6)	1 (5.9)	4 (6.8)	>0.99
Other	18 (23.7)	7 (41.2)	11 (18.6)	0.1
Primary Tumor Stage				
0	1 (1.3)	0	1 (1.7)	0.76
1	9 (11.8)	3 (17.6)	6 (10.2)	
2	20 (26.3)	4 (23.5)	16 (27.1)	
3	42 (55.2)	10 (58.8)	32 (54.2)	
4	4 (5.3)	0	4 (6.8)	
RT/CHT				
Neoadjuvant	20 (26.3)	2 (11.8)	18 (30.5)	0.2
Adjuvant	65 (85.5)	12 (70.6)	53 (89.8)	0.06
Number of Lesions				
1	61 (80.3)	10 (58.8)	51 (86.4)	0.013
2	11 (14.5)	5 (29.4)	6 (10.2)	
3	2 (2.6)	2 (11.8)	0	
4	1 (1.3)	0	1 (1.7)	
5	0	0	0	
6	1 (1.3)	0	1 (1.7)	
Bilateral nodules	7 (9.2)	1 (5.9)	6 (10.2)	>0.99
DFI	29 (IQR 17.5–45)	19 (IQR 12–38)	31 (IQR 18–45)	0.23

Values are expressed as *n* (%) or median (interquartile range). Abbreviations: ASA: American Society of Anesthesiologist Score, BMI: Body Mass Index, CHT: Chemotherapy, DFI: Disease-free Interval, IQR: Interquartile Range, RT: Radiotherapy.

**Table 2:** Operative approach and kind of resection.

Population	All	BMI ≥30	BMI <30	*p*
	76 (100)	17 (22.3)	59 (77.7)	
Resection				
Wedge Resection	39 (51.3)	6 (35.3)	33 (55.9)	0.17
Segmentectomy	3 (3.9)	1 (5.9)	2 (3.4)	0.54
Lobectomy	22 (40.8)	9 (52.9)	22 (37.3)	0.27
Segmentectomy + Wedge	1 (1.3)	0	1 (1.7)	>0.99
Lobectomy + Wedge	2 (2.6)	1 (5.9)	1 (1.7)	0.4
Approach				
VATS	35 (46.1)	5 (29.4)	30 (50.8)	0.16
Post-Resectional Status				
R0	74 (97.7)	17 (100)	57 (96.6)	>0.99
R+	2 (2.6)	0	2 (3.4)	

Values are expressed as *n* (%). Abbreviations: R0: No Residual Disease, R+: Presence of Residual Disease, VATS: Video-Assisted Thoracic Surgery.

**Table 3:** Complications and Survival.

Population	All	BMI ≥30	BMI <30	*p*
	76 (100)	17 (22.3)	59 (77.7)	
Length of stay	6 (4–11)	7 (5–14)	6 (4–11)	0.14
Complications				
Total	11 (14.5)	3 (17.6)	8 (13.6)	0.7
Haemorrhage	3 (3.9)	2 (11.8)	1 (1.7)	0.12
Persistent Air-leak	4 (5.6)	0	4 (6.8)	0.57
Arrhythmia	3 (3.9)	1 (5.9)	2 (3.4)	0.53
ARDS	1 (1.32)	0	1 (1.7)	>0.99
Pneumonia	5 (6.6)	1 (5.9)	4 (6.8)	>0.99
Other	4 (5.6)	1 (5.9)	3 (5.1)	>0.99
Survival and recurrence				
5 Years-OS		66.70%	33.60%	0.03
5 Years-DFS		36.20%	21.50%	0.75

Values are expressed as *n* (%) or median (interquartile range). Abbreviations: ARDS: Acute Respiratory Distress Syndrome, CSS: Cancer-Specific Survival DFS: Disease-Free Survival, OS: Overall Survival.
